# Conception, pregnancy and inflammatory bowel disease—Current concepts for the practising clinician

**DOI:** 10.1007/s12664-024-01563-9

**Published:** 2024-05-15

**Authors:** Eleanor Liu, Kelly Chatten, Jimmy K. Limdi

**Affiliations:** Department of Gastroenterology, Northern Care Alliance NHS Trust, Manchester, UK

**Keywords:** Fertility, Inflammatory bowel disease, Postpartum, Preconception, Pregnancy

## Abstract

The peak incidence of inflammatory bowel disease (IBD) coincides with a woman’s prime reproductive years. The management of IBD during pregnancy can be challenging for healthcare professionals, underpinning the need for a multi-disciplinary approach with shared decision-making with the patient. Pre-conception counselling can address patient concerns, improve pregnancy specific IBD patient knowledge and provide a personalized risk assessment, to ensure optimal maternal and fetal outcomes. Most women with IBD have fertility rates comparable with the general population, although voluntary childlessness is common among women with IBD. IBD disease activity at conception and during pregnancy is a key determinant of the course of IBD during pregnancy. Active IBD during pregnancy is associated with adverse pregnancy-related outcomes, including spontaneous abortion, small for gestational age baby and preterm birth, emphasizing the importance of ensuring disease remission prior to conception. Most IBD medications (5-aminosalicylates, thiopurines if already initiated pre-conception, corticosteroids and biologic medications) are considered safe and low risk during pregnancy and breastfeeding, except for methotrexate, JAK-inhibitors, ozanimod and allopurinol and maintaining remission throughout gestation should be the priority. Most women with IBD can have a vaginal delivery, but cesarean section should be considered in active perianal disease and history of ileal pouch surgery. This narrative review outlines the current evidence for the management of IBD in pregnancy, as well as considering the pre-conceptual and post-partum period.

## Introduction

The peak incidence of inflammatory bowel disease (IBD) is between the second and fourth decades of life, coinciding with a woman’s reproductive years [1]. The management of IBD in pregnancy can be challenging, but it is concerning that not only is patient knowledge on reproductive issues sub-optimal [2], knowledge among healthcare professionals also remains inconsistent [3], emphasizing the need for further patient and clinician education. This review article aims to provide an overview of the current literature on managing IBD in the pre-conception, pregnancy and post-partum period for the practising clinician.

## Preconception

Unintended or mistimed pregnancies are associated with a higher risk of delayed pre-conceptual care and adverse maternal and neonatal outcomes [4]. They account for nearly 50% of pregnancies, up to a half of which may be due to contraceptive failure, related to poor understanding and what contributes to highly effective methods [4]. In an American study, 23% of women with IBD of child-bearing age were not using any form of contraception, with only 17% using highly effective methods (namely the contraceptive implant, intrauterine devices and sterilization). Those not on highly effective contraception are at potentially greater risk of unplanned pregnancies [5].

Fertility is a concern for many IBD patients [6], although rates of infertility in inactive IBD with no previous pelvic surgery are comparable to those in the general population [7, 8]. Voluntary childlessness appears to be more frequent in women with IBD [6, 9], driven mainly by concerns around heritability and medication risk [8–10]. Most women with IBD in remission do not have compromized ovarian reserve (reduced quality and quantity of ovarian primordial follicular pool) compared to the general population, although ovarian reserve with active Crohn’s disease (CD) may be reduced [7, 11]. The only factor that affected fertility (excluding surgery, i.e. pouch) in women with ulcerative colitis (UC) was age > 35 years as a physiological reduction in ovarian reserve [9].

Ileal pouch anal anastomosis (IPAA) surgery has been associated with up to a threefold increased risk of infertility [12, 13]; thus, women of child-bearing age should be counselled regarding the risks of pelvic surgery and associated impact on fertility [8]. Average infertility rates were 20% pre-IPAA and 63% post-IPAA. The relative risk of infertility after IPAA is 3.91 ([2.06, 7.44] 95% CI) [13]. Erectile dysfunction following IPAA is also a recognized association [8]. Notably, the data is from the era of open laparotomy and outcomes from laparoscopic surgery are needed.

A majority of IBD medications do not impact fertility. Sulfasalazine can cause reversible male infertility by lowering sperm count and motility [8].

Regarding timing of referrals for fertility evaluations in women with IBD, a widely accepted approach is to refer after six months of failure to conceive in women ≥ 35 years or prior pelvic surgery or after 12 months of timed and unprotected intercourse in women under 35 years [14]. A recent survey of UK and Australian gastroenterology clinicians found that 70% had never initiated a fertility referral for IBD patients [15]. Assisted reproductive technology (ART), the commonest being in-vitro fertilization (IVF) or other modalities such as gamete intra-fallopian transfer, zygote intra-fallopian transfer or frozen embryo transfer may be an option in women with IBD [16]. However, over half surveyed were uncertain about the efficacy of ART in IBD patients and all participants believed they had low knowledge levels [15]. A systematic review concluded that women with UC, functioning IPAA (UC) and medically managed CD respond well to ART, with success rates comparable with the general population [16]. Success of ART was lower (49% to 71%) in surgically managed CD patients and 64% lower in patients with UC and IPAA failure [16].

### Genetic risk

Hereditability is a concern for many IBD patients and contributes to voluntary infertility. A family history of IBD has been reported in up to 12% of CD patients and 9% in UC, being higher (up to 33%) in children with multiple family members diagnosed with IBD [8, 18]. Other factors increasing risk include increasing number of affected relatives (specifically both parents), younger onset and certain ethnic groups such as Ashkenazi Jews [8, 17, 18].

### Pre-conception counselling

Disease activity in the pre-conception period is an important predictor of disease activity in pregnancy, affirming the importance of achieving disease remission at least three months pre-conception. Although international guidelines recommend that pre-conception counselling should be available to all women of child-bearing age with IBD [8, 14, 19–21], a recent UK survey found this was available in only 39% units [22]. Sub-optimal patient knowledge and uninformed patient decision-making contributes to voluntary infertility and medication discontinuation during pregnancy, increasing the risk of IBD flares and subsequently, adverse maternofetal outcomes [2, 21]. Psychosocial stigma around having IBD, use of advanced therapies and in some instances surgery (i.e. stoma or fistula) can be responsible for a negative impact on inter-personal relationships and voluntary childlessness. Pre-conception education improves healthier behaviors, in turn improving pregnancy outcomes [14, 19–21, 23]. Safety of IBD medications during pregnancy and lactation should be discussed with patients and potentially teratogenic agents (for example methotrexate, JAK inhibitors and S1P modulators) discontinued [8, 17, 19]. Disease activity should be assessed objectively and disease control optimized to achieve clinical, biochemical (by assessment of C-reactive protein [CRP] and fecal calprotectin) and if possible endoscopic remission prior to conception [8, 17, 19]. Women should be encouraged to ensure they are up to date with vaccinations and nutritional status optimized where appropriate. There is an association between IBD, immunosuppression, human papilloma virus and an increased risk of high-grade cervical dysplasia and cervical cancer. Compliance with cervical screening should be encouraged in women with IBD and UK guidelines advise IBD patients follow the standard national screening programme [20]. Alcohol intake, smoking cessation and recreational drug use should also be addressed in pre-conception counselling [8]. Iron, folic acid and B_12_ levels should be checked for deficiencies at conception. Planned pregnancy would enable folic acid supplementation to be commenced one month prior to conception and should be continued till the completion of the first trimester to reduce the risk of neural tube defects [14, 19]. Sulfasalazine inhibits folate synthesis; thus, these patients should supplement higher doses of folic acid (≥ 2 mg/day) [8, 14, 19]. Furthermore, for women with current corticosteroid use, a history of pre-gestational hypertension or diabetes, aspirin prophylaxis (75–150 mg daily), is recommended from 12 weeks of gestation and typically discontinued at week 36 [24].

## Management during pregnancy

### Impact of pregnancy on IBD

There is an increase in tumor necrosis factor (TNF) and other pro-inflammatory cytokines observed in successful pregnancy. It is believed that this increase in natural cytokines in pregnancy, combined with an immune-mediated condition such as IBD, can increase the risk of adverse maternal and fetal outcomes [14].

Unplanned or mistimed pregnancies are associated with a higher risk of delayed pre-conceptual care, increased risk of preterm birth, low birth weight (LBW) and adverse maternal and neonatal outcomes [25–27]. Furthermore, IBD activity during pregnancy is associated with adverse pregnancy-related outcomes such as miscarriage, intra-uterine growth retardation and preterm birth [28–30].

Disease activity at conception increases the likelihood of flare throughout pregnancy and post-partum [8, 14]. Pedersen et al. reported that while pregnant women with CD had a similar disease course both during pregnancy and post-partum as non-pregnant women, those with UC were at higher relapse risk during pregnancy and post-partum [26]. Rottenstreich et al. prospectively followed women from conception through to post-partum: 37.6% of the 298 women with quiescent disease at conception experienced a flare during pregnancy. The risk of disease relapse was higher in UC patients compared to CD (48.1% vs. 31.8%, *p* = 0.001) [31]. A systematic review and meta-analysis including 28 studies reported an association of IBD flares during the pre-conception and pregnancy period and a higher risk of pregnancy-related complications compared to patients with quiescent IBD with pooled odds ratios: LBW (OR 3.8 [95% CI; 1.8–8.0]), small for gestational age (OR 1.5 [95% CI; 1.2–1.9]), pre-term birth (OR 2.4 [95% CI; 1.7–3.4]), pre-eclampsia (OR 2.8 [95% CI; 0.7–11.6]), early pregnancy loss (OR 1.9 [95% CI; 1.2–3.0]) and stillbirth (OR 2.3 [95% CI; 1.0–5.0]) [27].

The risk of continued disease activity throughout pregnancy is nearly doubled among patients with active IBD at conception. Moreover, in patients who experienced an IBD flare during pregnancy, the risk of active IBD during subsequent pregnancies may be increased [25]. 

**Fig. 1:**
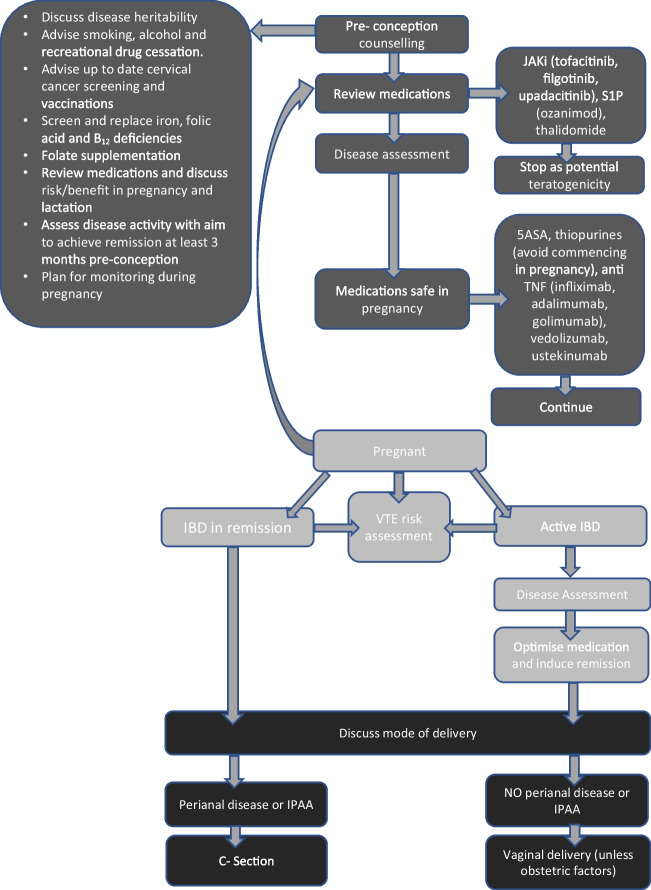
Overview of management of inflammatory bowel disease in pregnancy

Figure 1 provides an overview of the main principles to be considered in the management of IBD in pregnancy.

### Safety of medications

Safety of medications during pregnancy remains a priority for women and the most frequent reason for non-adherence is fear of medication-related risks to the fetus [2, 8]. It is important to counsel women with the best available data, considering the areas where data is lacking and provide reassurance that with treatment optimization, a majority will not experience disease-related complications during pregnancy (Table 1.).

**Table 1.** Overview of inflammatory bowel disease medications during pregnancy and lactation [8]

Medication	Use during pregnancy	Use during breast feeding
***Aminosalicylates***		
5-aminosalicylates	• Low risk • Maintain pre-pregnancy dose	• Low risk • Negligible amount transferred
Sulfasalazine	• Low risk • Folic acid supplementation ≥2 mg/day.	
***Immunomodulators***		
Thiopurines	• Low risk • Avoid commencing in pregnancy (unpredictable risk of adverse events) • Continue during pregnancy as monotherapy • Review the need to continue if being used as combination therapy with biologic medication • Consider monitoring metabolite levels during pregnancy	• Low risk • Very small amounts of metabolites in breastmilk
Methotrexate	• Contra-indicated- teratogenic • Stop 3-6 months prior to conception.	• Detectable in breastmilk • Not recommended in breastfeeding
***Antibiotics***		
	• Penicillin containing antibiotics preferred during pregnancy for IBD indications • Ciprofloxacin: animal studies reported musculoskeletal abnormalities; avoid especially in T1 • Metronidazole: Low risk, but some data suggesting cleft lip risk with T1 exposure.	• Penicillin containing antibiotics are safe in breastfeeding • Ciprofloxacin and metronidazole are excreted into breastmilk and should be avoided
***Corticosteroids***		
	• Low risk • Short courses only; reserve for active flares during pregnancy, not for maintenance therapy • Budesonide: low risk • Prednisolone: low risk, increased risk of gestational diabetes (consider growth scanning), potential increased risk of preterm birth, LBW, orofacial anomalies (but data confounded by disease activity).	• Low risk • Low concentrations found in breastmilk • Can consider 4 hour delay before feeding, but may not be practical
***Biologics***		
Anti-TNF	• Low risk • Maintain pre-pregnancy dosing • Exception is certolizumab (does not actively cross placenta); very low risk • Consider continuing throughout pregnancy, can adjust timing of last dose prior to delivery to minimize placental transfer, T2 trough levels can guide dosing dates • Can restart from 48 hours postpartum	• Low risk • Detected in breastmilk in very small amounts and inactivated by baby’s digestive enzymes, therefore not absorbed • No impact found on neonatal infections rates
Vedolizumab	• Limited data • Low risk • Can restart from 48 hours postpartum	• Low risk, limited data • Low levels detectable in breastmilk • Likely similar principles to anti-TNF
Ustekinumab	• Limited data • Low risk • Can restart from 48 hours postpartum	• Low risk • Low levels detectable in breastmilk • Likely similar principles to anti-TNF
***Small molecules***		
Tofacitinib	• Very limited data • Avoid during pregnancy • Animal studies suggested teratogenic and feticidal effects	• Insufficient data- avoid
Filgotinib Upadacitinib	• Very limited data • Avoid during pregnancy	• No data; avoid
Ozanimod	• Very limited data • Contraindicated	• No data; avoid

*mg* milligram, *T1* first trimester, *anti-TNF* anti-tumor necrosis factor, *LBW* low birth weight, *T2* second trimester

#### 5-ASA and sulfasalazine

5-ASA and sulfasalazine are considered safe during pregnancy and should be continued throughout gestation to maintain remission [8, 14, 19, 20]. There is no increased risk of congenital anomalies or adverse pregnancy outcomes with 5-ASA [32].

#### Thiopurines

Azathioprine use during pregnancy is considered low risk and continuation is advocated by international guidelines [8, 14, 19, 20]. A systematic review also found no increased risk of congenital anomalies or LBW, but did find an increased risk of preterm birth [33].

More recently, the Pregnancy in IBD Neonatal Outcomes  (PIANO) Registry included 1490 pregnancies with 242 cases thiopurine monotherapy exposure and 227 cases of combination biologic/thiopurine therapy. They found no adverse maternal or fetal outcomes following thiopurine exposure, including congenital malformations, spontaneous abortion, LBW, preterm birth or neonatal infections [34].

Although the evidence is reassuring for thiopurine continuation during pregnancy, its initiation in pregnancy is discouraged due to the risk of short-term side effects such as nausea, reduced appetite, myalgia, deranged liver function and in particular risk of pancreatitis (albeit low) which is associated with a higher pre-eclampsia risk [14, 35]. In hypermethylators, there is preferential metabolism of azathioprine to 6-methylmercaptopurine (6-MMP). The addition of allopurinol can be used in some instances to divert metabolism to thioguanine nucleotides (TGN). While this strategy should be used cautiously in non-pregnant IBD patients, combining allopurinol with low-dose thiopurine therapy in pregnancy is not advised, as allopurinol may pose a risk to the fetus [36].

#### Methotrexate

Methotrexate is teratogenic and contra-indicated during pregnancy [8, 14, 19]. Effective contraception is imperative and women should stop methotrexate three to six months prior to attempting to conceive [8, 17].

If a woman conceives while taking methotrexate, the drug should be stopped immediately and the patient should be counselled and referred to obstetrics for further management including fetal scanning [8, 17, 19].

#### Antibiotics

Antibiotics (commonly ciprofloxacin and metronidazole) are often used in the treatment of perianal Crohn’s, pouchitis and abdominal sepsis.

A cohort study of 922 women exposed to metronidazole during all trimesters showed no adverse outcomes including congenital malformations [37]. Animal studies have demonstrated musculoskeletal anomalies with quinolone exposure; however, human studies have not confirmed this [8]. The overall risk is minimal with short courses of metronidazole or ciprofloxacin, but it may be preferable to avoid first trimester use if alternative antibiotics such as penicillin (amoxicillin or co-amoxiclav) are available [8, 14].

#### Corticosteroids

Corticosteroid therapy is considered low risk during pregnancy, but should only be used to manage acute exacerbations and not for maintenance therapy [8, 19, 20]. The use of corticosteroids during pregnancy increases the risk of gestational diabetes (OR 4.3; 95% CI 1.2–16.3) [38]. Women receiving corticosteroid therapy during pregnancy should have regular glycemic monitoring and serial third trimester growth scanning [17, 19].

A population-based study of over 50,000 pregnancies with first trimester corticosteroid exposure failed to show any increased risk of orofacial malformations [39]. Of 432 pregnancies with maternal corticosteroid exposure in the PIANO registry, there was no significant difference in the rate of congenital malformations in the corticosteroid-exposed group (10%) vs. those unexposed (9%), *p* = 0.37 [40]. Corticosteroid exposure was associated with increased preterm birth risk (OR 1.79, 95% CI 1.18 to 2.73), LBW (OR 1.76, 95% CI 1.07 to 2.88) and neonatal intensive care unit (NICU) admission (OR 1.54, 95% CI 1.03 to 2.30) [40]. Notably, most data from studies looking at the risks of corticosteroid exposure are confounded by disease activity.

### Biological medications

#### Anti-TNF agents

Despite the growing body of evidence supporting the safety of anti-TNF agents, clinicians have had reservations in continuing anti-TNF agents throughout pregnancy. During the second and third trimester, IgG monoclonal antibody biologics are actively transported across the placenta and can be detected in infants up to nine months after birth, with the exception of certolizumab. Certolizumab lacks an Fc region, thus cannot bind to the neonatal Fc receptor in the placenta and therefore cannot cross the placental barrier [34].

Updated international guidelines advise that there is no increased risk of infant infection or maternofetal adverse outcomes with continuation beyond 30 weeks and anti-TNF agents should be considered for patients with active disease or high risk of relapse [14, 19, 20]. They advise adjusting the timing of the last dose to achieve lower trough levels at delivery, aiming for last infliximab dosing six to 10 weeks and adalimumab two to three weeks prior to delivery [14]. There may also be a role in checking second trimester anti-TNF drug levels and adjusting dosing schedules accordingly to reduce placental transfer [8, 14].

The PIANO registry [34] reported 869 pregnancies exposed to biologics (642 biologic monotherapy, 227 biologic/thiopurine combination therapy), wherein 97% received anti-TNF, 6% anti-integrin and 2% ustekinumab. Biologic and/or thiopurine exposure was not associated with increased risk of congenital anomalies, spontaneous abortions, preterm birth, LBW, neonatal infections or impaired developmental milestones. Higher maternal disease activity, however, was associated with spontaneous abortion risk (HR 3.4, 95% CI 1.5–7.7) and preterm birth with increased risk of neonatal infection (OR 1.7, 95 CI 1.2–2.5) [34].

The EVASION study, a large retrospective cohort study included 1456 anti-TNF exposed pregnancies in women with IBD and concluded no increased one-year neonatal infection rates, where anti-TNF was continued throughout pregnancy (an OR = 89; 95% CI 0.76–1.05) [41]. They did, however, find a higher rate of disease flare-up (46% if anti-TNF agents were discontinued before week 24, vs. 31% if anti-TNF agents continued beyond week 24), *p* = 0.005. In a systematic review and meta-analysis of 48 studies (6963 patients with IBD), receiving biologics in pregnancy, adverse outcomes (preterm birth, still birth, LBW and congenital malformation) were not higher in biologic-exposed pregnancies compared to the general population [42]. Continuation of therapy through third trimester was not associated with an increase in risks of adverse pregnancy outcomes vs. earlier discontinuation.

#### Vedolizumab, ustekinumab and small molecules

Data on the outcomes of pregnancy with vedolizumab and ustekinumab and Janus kinases (JAK) inhibitors is limited. Animal studies show no adverse effects from vedolizumab and ustekinumab exposure on prenatal or postnatal development [17]. The clinical trials programme for both drugs showed no safety concerns [43, 44].

The Groupe d’Etude Thérapeutique des Affections Inflammatoires Digestives (GETAID) group assessed 73 pregnancies exposed to ustekinumab or vedolizumab and compared outcomes to a control group of anti-TNF exposed pregnancies [45]. There were similar rates of preterm birth, miscarriage and congenital malformations across all groups [45]. Similarly, the CONCEIVE study reported consistent rates of miscarriage, preterm birth, LBW, congenital anomalies and neonatal infections, between the vedolizumab-exposed group, anti-TNF exposed group and non-biologic/immunomodulator exposed group [46].

A preliminary analysis from the DUMBO registry, assessing the safety and long-term outcomes of IBD drugs in mother and infants up to four years of age, reported that biologic monotherapy or combination therapy did not increase the risk of serious adverse events (AEs) during pregnancy (OR 0.8, 95% CI 0.2–3) [47].

Although the evidence to date suggests both ustekinumab and vedolizumab are safe during pregnancy, until more robust data was available, the patient should be counselled and the risks of withdrawing treatment vs. continuing discussed. If these agents are to be continued during pregnancy, it is advised that the same practice applies as for anti-TNF agents [8, 20].

#### Small molecules

There are limited safety data for the use of small molecules such as tofacitinib during pregnancy. As a small molecule, tofacitinib is likely to cross the placental barrier and be secreted in breast milk and animal studies have shown teratogenic and feticidal effects [48].

In a recent report of 1157 patients enrolled in the UC interventional studies with 11 cases of maternal exposure and 14 cases of paternal exposure to tofacitinib (5 mg or 10 mg twice daily) before or at the time of conception or during pregnancy, outcomes included 15 healthy newborns, no fetal or neonatal deaths, no congenital malformations, two spontaneous abortions and two medical terminations [49]. Data on newer JAK-inhibitors, filgotinib and upadacitinib is limited [50]. Sphingosine 1-phosphate (S1P) modulator (ozanimod) is involved in regulating events during embryogenesis such as angiogenesis, cardiogenesis, limb development and neurogenesis [51, 52].

The use of JAK inhibitors and S1P modulators in women planning pregnancy or during pregnancy and lactation is not advised [8] and effective contraception [4] is advised during treatment and up to six weeks after their use.

## Investigations during pregnancy

Pregnant patients showing clinical signs of active disease should be assessed with prompt treatment optimization to ensure remission is achieved and maintained. There are specific factors to take into consideration when investigating IBD in pregnancy, as discussed in the following and summarized in Table 2.

**Table 2:** Overview of role of investigations in managing inflammatory bowel disease during pregnancy

Investigation	Practical points
Fecal calprotectin	• Correlates well with endoscopic disease activity • Not affected by physiological changes in pregnancy • Non-invasive • Useful adjunctive tool to monitor disease activity during pregnancy
Blood parameters—hemoglobin, albumin, CRP	• Pregnancy physiological changes can alter these serum biomarkers • Do not correlate with clinical disease activity in pregnancy
Lower GI endoscopy	• Defer colonoscopies until T2 if possible • Flexible sigmoidoscopy safe throughout pregnancy, perform only if strong indication to guide clinical decision-making • Perform in left pelvic tilt or left lateral position • Minimise procedure time • Unsedated procedure preferable • Discuss plans with obstetric team
Intra-abdominal intestinal USS	• Safest imaging modality in pregnancy • Views of the bowel (particularly TI) limited from T3 onwards
MRI	• No radiation risk • Some concerns regarding foetal exposure to magnetic field, tissue heating effects and acoustic noise • Avoid gadolinium contrast in T1
CT	• Avoid if possible • If needed, use low radiation dose

*CRP* C-reactive protein, *GI* gastrointestinal, *USS* ultrasound scan, *MRI* magnetic resonance imaging, *CT* computed tomography, *T2* second trimester, *TI* terminal ileum, *T3* third trimester, *T1* first trimester

### Biochemical markers

Biochemical markers of disease activity can be useful adjuncts in assessing disease activity during pregnancy. Fecal calprotectin (FC) is a non-invasive surrogate marker of gut inflammation, correlating well with endoscopic disease activity and not affected by the physiological changes in pregnancy [8, 20, 53]. There is a growing body of evidence supporting FC as a useful adjunctive tool to monitor disease activity and aid risk stratification of IBD management in pregnancy [53]. A systematic review demonstrated pooled sensitivity of 85% and 75% specificity in FC for diagnosing active disease during pregnancy [53].

### Endoscopy

In certain circumstances, lower gastrointestinal endoscopy during pregnancy may be required to diagnose or stage IBD activity. Safety data on endoscopy during pregnancy, particularly in IBD patients, remain limited.

Current guidelines advise deferring colonoscopies until second trimester unless compelling indications exist on a case by case basis [8, 20, 54]. Flexible sigmoidoscopy is safe throughout pregnancy and can be performed if there is a strong indication and the results are likely to impact on clinical decision-making [14, 17, 19, 54]. In a study of 48 pregnant women with known or suspected IBD undergoing sigmoidoscopy, no AEs were reported at any stage in pregnancy [55].

Specific considerations for any endoscopy during pregnancy include procedure time, radiation exposure (such as in ERCP [endoscopic retrograde cholangiopancreatography]), sedative and bowel preparation [14, 19, 54]. Patients should be positioned in a left pelvic tilt or left lateral position to prevent vena cava compression, thus minimizing maternal hypotension and placental hypoperfusion [8, 14, 19, 54]. Unsedated endoscopy is preferable due to the potential risks of fetal sedation, namely respiratory depression and teratogenicity [8, 17]. Midazolam is the preferred sedative during pregnancy; however, postpartum women should be informed that midazolam is excreted in breast milk and it may be advisable to withhold breastfeeding for four hours following administration [17]. Fentanyl is considered safe with the added advantage of low bioavailability in breastmilk to the neonate thus not impacting on breastfeeding [17]. Dosage of sedation will be guided by clinical discretion. Plans for endoscopy should be discussed with the obstetric team and peri-procedural fetal monitoring may be appropriate in some cases [8, 14, 19].

### Imaging

Imaging with risk of ionizing radiation, magnetic fields and administration of contrast agents should only be performed in pregnant women if the risk of misdiagnosis of an IBD-related complication outweighs the risks of the test [17, 19].

Ultrasound (US) and magnetic resonance imaging (MRI) are deemed the safest imaging modalities in pregnancy [8]. Intestinal ultrasound scans (USS) can identify active IBD throughout pregnancy with 84% sensitivity and 98% specificity, offering a reliable non-invasive option [56]. From third trimester, the fetus may limit views of the bowel, particularly the terminal ileum [19, 56].

Despite the lack of radiation risk with MRI, safety has not been established regarding fetal exposure to a magnetic field, tissue heating effects and high acoustic noise levels. The use of gadolinium contrast should be avoided during first trimester as free gadolinium ions may accumulate in amniotic fluid and enter the fetal circulation [8, 17]. In the absence of safety data, the fetal risk of gadolinium remains unknown and a gadolinium-free MRI protocol can be reliable in assessing pregnant patients with IBD [57].

Computed tomography (CT) should ideally be avoided; however, if deemed necessary, it is acceptable during pregnancy as the radiation exposure of one CT scan is unlikely to have an adverse effect to the fetus [8, 17, 19].

### IBD surgery during pregnancy

The risk of severely active IBD is a greater risk to the fetus than considered surgical intervention. As such, indications for urgent surgery in pregnancy should be the same as those for the non-pregnant women [8, 14, 19]. Indications include severe UC not responding to medical therapy, intestinal obstruction, perforation, hemorrhage, abscess or severe disease activity not responding to medical therapy [8, 14]. A temporary ileostomy may reduce the risk of post-operative complications after primary anastomosis [14].

In a recent review of 44 IBD patients who underwent surgery during pregnancy (59% for intestinal obstruction, 23% sub-total colectomy, 18% other), a small number of miscarriages and stillbirths occurred in all trimesters: 14% of surgeries in second trimester and 65% in third trimester ended with a simultaneous C-section (CS) or vaginal delivery. Of 40 neonates, 61% were premature, 47% had LBW and 42% needed hospitalization in the context of prematurity, neonatal sepsis and respiratory distress [58]. A systematic review of surgical IBD management during pregnancy noted that surgical intervention during third trimester universally resulted in the onset of labor and a near 50% preterm delivery rate [59].

## Obstetric considerations

The IBD and obstetric team should carefully consider individual risk factors to achieve optimal care [8, 14, 21]. The obstetric team should consider risk factors that can affect fetal growth such as disease activity, medications, comorbidities, age and smoking, to determine the need for serial growth scans in IBD patients [8, 21, 60]. High-risk patients with active IBD should have additional growth scans at, for example, 30 and 36 weeks [21, 60].

IBD is a known risk factor for venous thromboembolism (VTE) and pregnancy further increases this risk, with the highest risk observed six weeks post-partum [4, 8, 21, 61]. A meta-analysis of VTE during pregnancy and puerperium found the VTE risk during pregnancy to be twofold higher in women with IBD than in non-IBD controls (pooled RR 2.13), with an even higher risk post-partum (pooled RR 2.6) [61].

Low molecular weight heparin (LMWH) is safe and effective in preventing and treating VTE and has been used widely in pregnancy [8]. All pregnant IBD patients should be risk assessed for VTE and prophylactic LMWH considered in those with active disease, hospitalized and/or following CS [8, 14, 19, 21].

### Delivery

The decision regarding mode of delivery should be made early during pregnancy by the obstetric team in discussion with the mother, with advice from a gastroenterologist and/or colorectal surgeon on IBD-related factors that might influence mode of delivery.

Vaginal delivery carries a lower risk of complications than CS in the general population [62]. Most IBD patients may have a vaginal delivery except in active perianal disease or when IPAA is present [8, 14, 19, 63].

Active perianal disease appears to be associated with increased risk of post-partum perianal flares after vaginal delivery [63]. A population-based study from the US found 4th degree perianal laceration similar between patients without CD and those with CD without perianal disease. However, perianal disease was independently associated with higher rates of 4th degree laceration (OR 10.9 95% CI 8.3–4.1 *p* < 0.001) [64].

Current international guidance suggests that IPAA is a relative indication for a CS [8, 14, 21]. A meta-analysis from 2007 concluded no significant differences in pouch function after vaginal delivery [65] and a Canadian study found that although increased stool frequency and incontinence was reported during pregnancy, a majority (83%) returned to pre-pregnancy state postpartum [66]. Notably, sphincter, integrity and manometric pressures have been noted to be more frequently affected by vaginal delivery in patients with IPAA [67]. Considering that these women may already have borderline continence, recent guidance suggests that CS should be considered to minimize the risk of anal sphincter injury [19, 21].

The presence of a stoma in IBD is not a contra-indication for vaginal delivery [14, 17]. However, in a recent retrospective study of 82 pregnancies in IBD patients with stomas found, overall CS rate was 73%, significantly higher than that of the general population and IBD patients without stomas [68]. The reasons for the high CS rate are not clear; only 1/3 of cases had a clear indication documented for elective CS [68].

## Postpartum

### Post-partum disease activity

Active disease in third trimester (OR 6.3; 95% CI 2.8–17.3), therapy de-escalation during pregnancy (OR 3.0; 95% CI 1.0–8.7) and de-escalation post-partum (OR 4.43; 95% CI 1.55–12.65) are associated with a higher risk of flares [69]. A systematic review and meta-analysis found similar rates of post-partum active disease in CD and UC and again third trimester discontinuation of biological therapy and biological de-escalation after delivery were risk factors for flaring post-partum [70]. Complicated CD, specifically stricturing (OR 3.64 95% CI 1.31–10.08) and penetrating phenotype (OR 4.25 95% CI 1.82–13.23), were associated with increased risk of post-partum disease [70].

### Breastfeeding

In addition to the health benefits that breastfeeding confers all neonates such as complete nutrition and maternal immunoglobulins contributing to the baby’s immune system, there are added IBD-related benefits [17]. Breastfeeding in infancy is protective against the development of IBD and does not increase the risk of post-partum disease flares [18, 69]. Several mechanisms are thought to reduce the risk of developing IBD including influence on intestinal microbiome, passive transfer of immunoglobulins and the components of breastmilk interaction with the neonatal intestinal microbiome [71]. A systematic review and meta-analysis found that ever being breastfed was associated with a lower risk of CD (OR 0.71, 95% CI 0.59–0.85) and UC (OR 0.78, CI 0.67–0.91) in the neonate. This benefit was dose-dependent, with greater protection from 12 months of breastfeeding, than three or six months [71].

In general, the safety data for breastfeeding is strong for most drugs and women should be encouraged to continue medications to prevent risk of disease flares, with the exception of methotrexate, allopurinol and thalidomide and newer small molecules (JAK inhibitors and S1P receptor agonists) due to a lack of evidence [8, 14, 21, 72]. IBD medications that are safe during pregnancy are also safe during lactation. Table 1. provides an overview of use of drugs during lactation.

### Neonatal and infant vaccinations

Vaccinations are essential for the newborn, to prevent serious infections. Most vaccines are non-live and do not increase risk of viral reactivation, as such may safely be offered to infants exposed to biologics in vitro. Anti-TNF’s can be detected in the infant up to six to nine months after birth, mandating caution with live vaccines [8, 14, 19]. Despite the reassuring evidence available, including the PIANO registry, where no increased risk of neonatal infections up to 12 months was observed after maternal biologic exposure [34], there has been one fatal case of disseminated Bacillus Calmette-Guerin infection after in utero exposure to infliximab [73]. It is therefore recommended that infants with in utero third trimester biologic exposure (except certolizumab as it does not cross the placenta) should not receive live vaccinations until at least six months of age [8, 14, 19]. Table 3 provides an overview of live and non-live vaccines.

**Table 3:** Overview of live and non-live vaccines

Non-live vaccines	Live vaccines
*Vaccination strategies for non-live vaccines should not differ in infants exposed *in utero* to biologics from unexposed infants*	*Avoid in the first six months if exposed to biologics *in utero
Diphtheria/tetanus/pertussis	BCG (*Bacille Calmette-Guérin)*
Intra-muscular polio	Rotavirus*
* Hemophilus influenzae*	Measles, mumps, rubella (MMR)
Hepatitis B	Oral polio
Meningococcus	Intra-nasal influenza
Pneumococcal	Varicella zoster
Human papilloma virus	Yellow fever
Inactivated influenza	Oral typhoid
	Small pox
	Yellow fever

^*^In practice, unlikely to be given as no benefit when given after six months of age

In conclusion, the pregnancy and childbirth are important life events. Optimal control of IBD prior to conception is key for good outcomes in pregnancy as is keeping the mother well and in remission during pregnancy through optimization of medical therapy and general health maintenance. Education on medication, safety and disease risk is crucial as most immunomodulator and biological therapies (with the exception of methotrexate, thalidomide, allopurinol, small molecules-JAK-inhibitors and S1P modulators) may be continued through pregnancy and lactation. Except for obstetric indications that require a cesarean section and cases of perianal CD and patients with an IPAA, most pregnant women with IBD can have vaginal deliveries. No live vaccinations should be administered to the infant exposed to biological therapies during pregnancy. Liaison with allied health professionals such as IBD nurse/practitioner or clinical psychologist as necessary is invaluable at all stages in the management of the pregnant woman with IBD. The management of pregnant women with IBD exemplifies the virtues of personalized medicine and multi-disciplinary care, achieving the best possible outcomes for mother and baby.

## Data Availability

Not applicable as review article.

## Declarations

### Disclaimer

The authors are solely responsible for the data and the contents of the paper. In no way, the Honorary Editor-in-Chief, Editorial Board Members, the Indian Society of Gastroenterology or the printer/publishers are responsible for the results/findings and content of this article.
